# Acute myeloid leukemia and transcription factors: role of erythroid Krüppel-like factor (EKLF)

**DOI:** 10.1186/1475-2867-12-25

**Published:** 2012-06-07

**Authors:** Rosa Ayala, Joaquin Martínez-López, Florinda Gilsanz

**Affiliations:** 1 Department of Medicine, Universidad Complutense de Madrid, Madrid, Spain; 2 Hematology Division, Hospital Universitario 12 de Octubre, Avenida Córdoba s/n, 28041, Madrid, Spain

**Keywords:** Acute myeloid leukemia, Transcription factors, EKLF, GATA1, GATA2, cMPL; FLT3, NPM1, CEBPA mutations

## Abstract

We have investigated the role of erythroid transcription factors mRNA expression in patients with acute myeloid leukemia (AML) in the context of cytogenetic and other prognostic molecular markers, such as FMS-like Tyrosine Kinase 3 (FLT3), Nucleophosmin 1 (NPM1), and CCAAT/enhance-binding protein α (CEBPA) mutations. Further validation of Erythroid Krüppel-like Factor (EKLF) mRNA expression as a prognostic factor was assessed.

We evaluated GATA binding protein 1 (GATA1), GATA binding protein 2 (GATA2), EKLF and Myeloproliferative Leukemia virus oncogen homology (cMPL) gene mRNA expression in the bone marrow of 65 AML patients at diagnosis, and assessed any correlation with NPM1, FLT3 and CEBPA mutations. **EKLF-positive AML** was associated with lower WBC in peripheral blood (P = 0.049), a higher percentage of erythroblasts in bone marrow (p = 0.057), and secondary AMLs (P = 0.036). High expression levels of EKLF showed a trend to association with T-cell antigen expression, such as CD7 (P = 0.057). Patients expressing EKLF had longer Overall Survival (OS) and Event Free Survival (EFS) than those patients not expressing EKLF (median OS was 35.61 months and 19.31 months, respectively, P = 0.0241; median EFS was 19.80 months and 8.03 months, respectively, P = 0.0140). No correlation of GATA1, GATA2, EKLF and cMPL levels was observed with FLT-3 or NPM1 mutation status. Four of four CEBPA mutated AMLs were EKLF positive versus 10 of 29 CEBPA wild-type AMLs; three of the CEBPA mutated, EKLF-positive AMLs were also GATA2 positive. There were no cases of CEBPA mutations in the EKLF-negative AML group. In conclusion, we have validated EKLF mRNA expression as an independent predictor of outcome in AML, and its expression is not associated with FLT3-ITD and NPM1 mutations. EKLF mRNA expression in AML patients may correlate with dysregulated CEBPA.

## Background

Functional disturbance of transcription factors involved in normal erythroid and megakaryocytic development, such as GATA binding protein 1 (GATA1), GATA binding protein 2 (GATA2), Erythroid Krüppel-like Factor (EKLF) and Myeloproliferative Leukemia virus oncogen homology (c-MPL), is frequent in acute myeloid leukemia (AML), but the role of these disturbances in AML subtypes is unknown.

EKLF is a transcription factor that plays a critical role in erythroid cell differentiation. It activates adult hemoglobin through a highly conserved CACCC motif, known to mutate in human beta-thalassemias [1], and restricts megakaryocytic differentiation to the benefit of erythroid differentiation [2]. The EKLF gene dosage is crucial for regulating the surface phenotype and molecular identity of maturing primitive erythroid cells [3]. GATA1 expression is particularly important for proper differentiation and maturation of erythroid cells and megakaryocytes [4]. GATA1-null cells stall at the pro-erythroblast stage, after which they readily undergo apoptosis [5]. GATA2, another member of the GATA family, plays essential roles in hematopoietic stem cell and progenitor cell compartments during normal hematopoiesis. GATA2 also contributes to cell cycle progression, and the maintenance of megakaryocyte identity in GATA1-deficient cells. Moreover, overexpression of GATA2 facilitates aberrant megakaryopoiesis [6]. cMPL is an active player in early hematopoiesis [7] from the first stages of definitive hematopoiesis establishment through to the direct regulation of the expression of transcription factors or genes important in the self-renewal, proliferation and apoptosis of hematopoietic stem cells [8]. Transcription factor acute myeloid leukemia 1 (AML1)/Runt-related transcription factor 1 (Runx1) regulates the c-MPL promoter, both positively and negatively, by changing the binding partner according to cell type [9].

AML is characterized by heterogeneity and, despite recent progress, still remains a highly fatal disease. The most important outcome predictor in AML is cytogenetic findings. However, treatment stratification is still not ideal, particularly in the case of cytogenetically normal AML (CN-AML, 40%-50% of cases). In CN-AML, molecular markers such as aberrations in the nucleophosmin (NPM1) and FMS-related tyrosine kinase 3 (FLT3) genes have been shown to be clinically relevant. NPM1 mutation is classified as a primary genetic lesion (so-called class II mutation) that impairs hematopoietic differentiation [10]. The tyrosine kinase receptor, FLT3, is expressed in early hematopoietic progenitor cells and mediates important functions for proliferation and survival. Mutations in the FLT3 gene are found in many AML subtypes and are considered class I mutations conferring a proliferation and/or survival advantage of the leukemic blasts [10]. In the presence of the FLT3 mutation, expression and function of several myeloid transcription factors are significantly repressed, in contrast to the induction caused by the activation of wild-type FLT3. These results indicate that FLT3 mutations induce aberrant receptor function which influence, not only proliferation and survival, but also the myeloid differentiation program [11].

The transcription factor CCAAT enhancer binding protein alpha (CEBPA) [12] is crucial for normal development of granulocytes and there is evidence that impaired CEBPA function contributes directly to the development of AML [13]. Various mechanisms have been identified as to how CEBPA function is dysregulated in patients with acute myeloid leukemia (AML). Mutations either in combination on separate alleles, or as sole mutations, or through promoter methylation by DNA methyltransferase, have been described [14]. In the absence of a co-existent FLT3-ITD (internal tandem duplication), a CEBPA mutation confers a significantly better prognosis in patients with CN-AML [15], with approximately 60% long-term survival, particularly if a “double” (bi-allelic) mutation is found [16].

We previously reported EKLF to be a favorable prognostic marker in AML. Higher EKLF expression was found in secondary AMLs versus primary AMLs, and patients expressing EKLF had longer overall survival (OS) and event-free survival (EFS), than those without EKLF [17]. However, no relationship between the dysregulation of erythroid transcription factors in AML and the presence of NPM1 and FLT3 mutations has been reported. Nevertheless, mutations of the CEBPA gene are associated with the upregulation of several genes involved in erythroid differentiation [18].

As CEBPA mutations are associated with the upregulation of genes involved in erythroid differentiation in a subtype of AML with higher hemoglobin levels and better outcome, we speculate that the AML subtype defined by EKLF expression may be generated via CEBPA silencing. We aim to validate EKLF expression as a favorable prognostic predictor in the context of other molecular markers in cytogenetically normal (CN) acute myeloid leukemia (AML).

## Results

### GATA1, GATA2, EKLF, and cMPL mRNA expression

**GATA1 expression** was detected in 35 cases and the average, median (range) of expression was 3.98, 0.0569 (0.006-176). **GATA2 expression** was detected in 40 cases and the average, median (range) of expression was 849.74, 0.2724 (0.003-39913.4). **EKLF expression** was detected in 35 cases and the average, median (range) of expression was 15.49, 0.1770 (0.0004-772.0). **cMPL expression** was detected in 46 cases and the average, median (range) of expression was 10.25, 0.8391 (0.01-132.9).

EKLF levels in the AML samples had significant positive correlations with levels of GATA1 (Rho 0.298, P = 0.008); GATA2 (Rho 0.263, P = 0.017), and cMPL (Rho 0.230, P = 0.033). There was a tendency to a positive correlation between levels of GATA1 and GATA2 (Rho 0.188, P = 0.067).

### Characteristics of AML patients expressing GATA1, GATA2, EKLF, and cMPL

**Figure 1:**
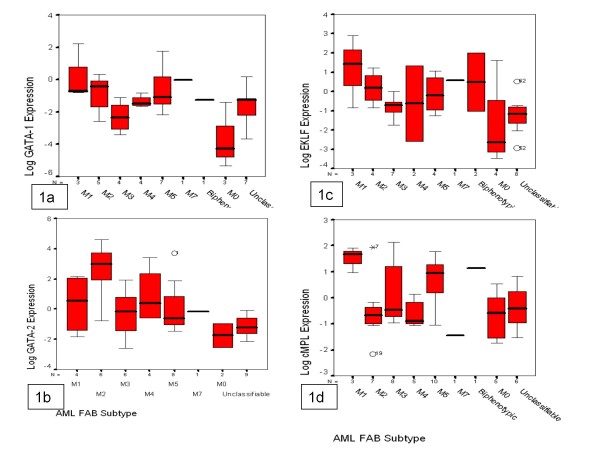
**Boxplot of GATA1 (1a), GATA2 (1b), EKLF (1c), and cMPL (1d) mRNA expression of in different AML subtypes.** EKLF expression was found to have no significant difference among French American British (FAB) classes. GATA-1, GATA-2 and CMPL expression were significantly different among FAB classes (P = 0.043; P = 0.007 and P = 0.025, respectively). GATA binding protein 1 (GATA1), GATA binding protein 2 (GATA2), Erythroid Krüppel-like Factor (EKLF) and Mieloproliferative Leukemia virus oncogen homology (c-MPL).

The characteristics of **GATA1-positive AML** patients were: male (P = 0.011) and older age (P = 0.011). They showed frequent co-expression of EKLF (24 of 35 GATA1 positives versus 11 of 30 GATA1 negatives, P = 0.010), and median EKLF expression was higher in this subtype than GATA1-negative AML patients (27.4 versus 1.6, P = 0.019) (Table 1). GATA1 expression was significantly different among FAB classes (P *=* 0.043) (Figure 1a).

**Table 1:** Comparison of clinical and biological characteristics between acute myeloid leukemia (AML) patients, with or without GATA-1 and GATA-2 expression

	**Gata-1 positive**	**Gata-1 negative**	**P**	**Gata-2 positive**	**Gata-2 negative**	**P**
Gender of patients (male/female)	25/10	12/18	**0.011**	25/15	12/13	0.251
Age/years (median)	57.3	42.2	**0.011**	52.7	44.7	0.187
Status Performance/ECOG	6/11/12/6/0	11/7/8/3/1	0.308	10/13/11/5/1	7/5/9/4/0	0.728
WBC count, x10^9^/L(median)	34.98	34.34	0.965	30.6	41.1	0.473
Hemoglobin, g/dL (median)	9.3	9.8	0.303	9.8	9.3	0.168
platelet count, x10^9^/L (median)	103.4	75.4	0.219	102.2	73.6	0.217
Percentage of Blasts in PB (median)	36.7	49	0.153	42.8	41.6	0.885
M2 vs. non-M2 FAB	5/29	6/24	0.575	6/33	5/20	0.633
M3 vs.non-M3 FAB	4/30	5/24	0.536	6/33	3/21	0.751
WHO Type	8/9/2/14	6/7/1/15	0.885	9/10/2/17	5/6/1/2	0.980
Primary/Secondary LMA	25/10	26/4	0.136	31/9	20/5	0.811
Cytogenetic (MRC)	8/16/7	7/15/5	0.924	10/20/5	5/11/7	0.329
Cr 7 Alterations (yes/no)	3/28	4/24	0.585	1/35	6/17	**0.007**
Cr 5 Alterations (yes/no)	2/29	5/23	0.176	2/34	5/18	0.061
Cr 11 Alterations (yes/no)	5/26	6/22	0.602	3/33	8/15	**0.011**
CD34 Blasts (yes/no)	19/16	14/15	0.820	20/19	13/12	0.955
CD117 Blasts (yes/no)	25/10	16/13	0.177	25/14	16/9	0.993
CD15 Blasts (yes/no)	16/15	15/13	0.880	17/19	14/9	0.306
CD11B Blasts (yes/no)	10/21	8/18	0.945	12/24	6/15	0.709
CD11C Blasts (yes/no)	8/1	7/3	0.313	5/7	10/2	0.539
CD 133 Blasts (yes/no)	11/16	9/11	0.770	12/17	8/10	0.836
CD 123 Blasts (yes/no)	6/14	6/9	0.537	4/18	8/5	**0.009**
HLA DR + Blasts (yes/no)	21/7	13/14	**0.040**	23/11	11/10	0.258
CD56 Blast (yes/no)	5/30	6/24	0.540	6/34	5/20	0.601
T-cell markers Blast (yes/no)	5/13	4/18	0.470	5/15	4/16	0.705
Erythroblasts BM	1/8/12	1/12/9	0.359	1/10/11	1/10/10	0.335
Gata-1 Expression (yes/no)				25/15	10/15	0.077
Gata-2 Expression (yes/no)	25/15	10/15	0.077			
EKLF Expression (yes/no)	24/11	11/19	**0/010**	27/13	8/17	**0.005**
cMPL Expression (yes/no)	26/9	20/10	0.501	31/9	15/10	0.131
Gata-1 (Median Expression)^*^				6.37	0.14	0.090
Gata-2 (Median Expression)	253.1	1545.85	0.182			
EKLF (Median Expression)	27.4	1.6	**0.019**	20.4	7.6	**0.017**
cMPL (Median Expression)*	9.2	11.5	0.780	11.97	7.50	0.181
Mutated NPM1 (yes/no)	4/20	5/15	0.495	6/22	3/13	0.832
Mutated FLT3 (yes/no)	2/22	7/13	**0.029**	6/22	3/13	0.832

PS, Performance Status. WBC, white blood cell. FAB indicates French American British classification [19] and WHO indicates World Health Organization classification [20]. MRC, cytogenetic classification of Medical Research Cancer [21]. GATA binding protein 1 (GATA1), GATA binding protein 2 (GATA2), Erythroid Krüppel-like Factor (EKLF) and Mieloproliferative Leukemia virus oncogen homology (c-MPL). * All expression ratios are given as 100x target gene/GUS.

The characteristics of **GATA2-positive AML** patients were: no alterations in chromosome 7 (P = 0.007), chromosome 11 (P = 0.011) or chromosome 5 (P = 0.065). They showed frequent co-expression of EKLF (27 of 40 GATA2 positives versus 8 of 25 GATA2 negatives, P = 0.005) and the expression median of EKLF was higher (20.4 versus 1.6, P = 0.017) (Table 1). GATA2 expression was found to be significantly different among FAB classes (P = 0.007)(Figure 1b).

The characteristics of **EKLF-positive AML** patients were: lower WBC in peripheral blood (median 21.05 versus 51.15, P = 0.049), a higher percentage of erythroblasts in bone marrow (median P = 0.057), and higher frequency of secondary AMLs (11 of 35 EKLF positives versus 3 of 30 EKLF negatives, P = 0.036). They showed frequent co-expression of GATA1 (24 of 35 EKLF positives versus 11 of 30 EKLF negatives, P = 0.010), and GATA2 (27 of 35 EKLF positives versus 13 of 30 negatives, P = 0.005), and the expression median of GATA1 was higher (7.28 versus 0.12, P = 0.038). A tendency to co-express cMPL (28 of 35 EKLF positives versus 18 of 30 EKLF negatives, P = 0.077) was observed (Table 2). No differences were found between FAB groups (Figure 1c). AML patients with high expression levels of EKLF tended to express T-cell markers such as CD7 (5 of 12 high-expression EKLF versus 4 of 28 low- or no expression EKLF, P = 0.057).

**Table 2:** Comparison of clinical and biological characteristics between acute myeloid leukemia (AML) patients, with or without EKLF and C-MPL expression

	**Eklf positive**	**Eklf negative**	**P**	**cMpl positive**	**cMpl negative**	**p**
Gender of patients (male/female)	22/13	15/15	0.297	25/21	12/7	0.514
Age/years (median)	46.0	50.6	0.463	46.2	53.8	0.237
Status Performance/ECOG	8/13/10/4/0	9/5/10/54	0.359	14/13/13/5/1	3/5/7/4/0	0.581
WBC count,x10^9^/L (median)	21.05	51.15	**0.049**	22.4	63.7	**0.006**
Hemoglobin, g/dL (median)	9.5	9.5	0.920	9.6	9.2	0.396
Platelet count, x10^9^(median)	107.0	71.1	0.114	84.3	106.1	0.388
Percentage of Blasts in PB (median)	38.3	47.5	0.290	37.2	53.4	0.077
M2 vs.non-M2 FAB	4/30	7/23	0.221	7/39	4/14	0.504
M3 vs.non-M3 FAB	7/28	2/26	0.147	8/37	1/17	0.210
M5 vs.non-M5 FAB	4/30	8/22	0.127	10/36	2/16	0.327
WHO Type	9/10/3/12	11/12/1/20	0.140	11/12/1/20	3/4/2/9	0.451
Primary/Secondary LMA	24/11	27/3	**0.036**	37/9	14/5	0.547
Cytogenetic (MRC)	10/16/6	5/15/6	0.580	11/22/9	4/9/3	0.961
Cr 7 Alterations (yes/no)	5/27	2/25	0.331	4/39	3/13	0.318
Cr 5 Alterations (yes/no)	2/30	5/22	0.147	5/38	2/14	0.927
Cr 11 Alterations (yes/no)	6/26	5/22	0.982	7/36	4/12	0.444
CD34 Blasts (yes/no)	19/16	14/15	0.632	24/21	9/10	0.663
CD117 Blasts (yes/no)	22/13	19/10	0.825	29/16	12/7	0.922
CD15 Blats (yes/no)	12/19	19/9	**0.025**	19/22	12/6	0.150
CD11B Blasts (yes/no)	5/25	13/14	**0.011**	13/28	5/11	0.973
CD11C BLasts (yes/no)	5/0	10/4	0.179	10/2	5/2	0.539
CD133 BLasts (yes/no)	10/18	10/9	0.250	16/19	4/8	0.454
CD123 Blasts (yes/no)	5/16	7/7	0.110	6/18	6/5	0.087
HLA DR + BLasts (yes/no)	19/9	15/12	0.348	26/14	8/7	0.428
CD56 Blasts (yes/no)	6/29	5/25	0.959	7/39	4/15	0.568
T-cell markers Blasts (yes/no)	5/11	4/20	0.279	3/23	6/8	**0.024**
Erythroblasts BM	2/6/11	0/14/10	0.057`	1/12/17	1/8/4/3	0.460
Gata-1 Expression (yes/no)	24/11	11/19	**0.010**	26/20	9/10	0.501
Gata-2 Expression (yes/no)	27/8	13/17	**0.005**	31/15	9/10	0.131
EKLF Expression (yes/no)				28/18	7/12	0.077
cMPL Expression (yes/no)	28/7	18/12	**0.007**			
Gat-1 (Median Expression)*	7.28	0.12	**0.038**	5.56	0.12	0.761
Gat-2 (Median Expression)*	1178.01	466.75	0.096	1009.84	462.12	0.552
EKLF (Median Expression)*				19.54	113.68	0.096
cMPL(Median Expression)*	10.9	9.4	0.174			
Mutated NPM1 (yes/no)	3/20	6/15	0.202	4/25	5/10	0.128
Mutated FLT3 (yes/no)	4/19	5/16	0.598	4/25	5/10	0.128

PS, Performance Status. WBC, white blood cell. FAB indicates French American British classification [19] and WHO indicates World Health Organization classification [20]. MRC, cytogenetic classification of Medical Research Cancer [21]. GATA binding protein 1 (GATA1), GATA binding protein 2 (GATA2), Erythroid Krüppel-like Factor (EKLF) and Mieloproliferative Leukemia virus oncogen homology (c-MPL). * All expression ratios are given as 100x target gene/GUS.

The characteristics of **cMPL-positive AML** patients were: lower WBC in peripheral blood (median 22.4 versus 63.7, P = 0.006); a lower percentage of blasts in peripheral blood (median 37.2 versus 53.4, P = 0.077). They showed a tendency to co-express EKLF, but not GATA1 or GATA2 (Table 2). cMPL expression was found to be significantly different among FAB classes (P = 0.016); cMPL levels were higher in FAB type M5 (P = 0.025) (Figure 1d).

In the multivariable analysis **GATA1 expression** was associated with EKLF expression (no/yes) (HR 4.69; 95%CI 1.48-14.82, P = 0.008), and patient age (continuous variable) (HR 1.04; 95%CI 1.01-1.07, P = 0.005). **GATA2 expression** was associated with EKLF expression (no/yes) (HR 13.32; 95%CI 2.80-63.35, P = 0.001), patient age (continuous variable) (HR 1.04; 95%CI 1.001-1.079, P = 0.04), and AML without chromosome 7 alterations (no/yes) (HR 0.02; 95% CI 0.001-0.25, P = 0.002). **EKLF expression** was associated with secondary AML (primary/secondary) (HR 10.71; 95%CI 1.54-74.49, P = 0.02), GATA2 expression (no/yes) (HR 5.45; 95% CI 1.5-19.68, P = 0.009), and cMPL expression (no/yes) (HR 5.16; 95%CI 1.51-23.08, P = 0.03). **cMPL expression** was associated with low WBC in peripheral blood (continuous variable) (HR 0.99; 95%CI 0.97-0.99, P = 0.04).

### FLT-3 or NPM1 mutations and correlations with GATA1, GATA2, EKLF and cMPL mRNA expression

NPM1 exon 12 mutations were detected in 20.5% of the studied AML samples, and FLT3 mutations were detected in 18.2%.

**NPM1 mutations** were associated with a high percentage of blasts in peripheral blood (79.7 versus 33.1, P < 0.001), a high percentage of bone marrow blasts (91.2 versus 63.5, P < 0.001) and *de novo* AML (100% *de novo* AML with NPM1 mutations versus 71.43% *de novo* AML without NPM1 mutations, P = 0.068). No association with GATA1, GATA2, EKLF, and cMPL expression were shown. No correlation was observed between GATA1, GATA2, EKLF and cMPL levels and NPM1 mutation status (Table 3).

**Table 3:** Comparison of clinical and biological characteristics between acute myeloid leukemia (AML) patients, with or without NPM1 and FLT3-ITD mutations

	**Mutated NPM1**	**WT NPM1**	**p**	**ITD FLT3**	**WT FLT3**	**p**
Gender of patients (male/female)	7/2	19/16	0.201	5/3	21/5	0.828
Age/years (median)	56.0	50.6	0.560	52.0	52.3	0.975
Status Performance/ECOG	3/3/1/2	9/7/15/4	0.347	2/4/1/1	10/6/15/5	0.190
WBC count,x10^9^/L (median)	109.60	17.14	**0.001**	80.04	26.28	**0.051**
Hemoglobin, g/dL (median)	9.59	9.35	0.724	9.46	9.39	0.893
Platelet, count, x10^9^/L (median)	58.0	94.4	0.332	60.87	93.64	0.142
Percentage of Blasts in PB (median)	79.7	33.1	**<0.001**	66.38	27.78	0.013
Percentage of Blasts in BM (median)	91.2	63.5	**<0.001**	80.29	66.69	0.132
M2 vs. non-M2 FAB	1/8	8/27	0.436	3/5	6/30	0.186
M3 vs. non-M3 FAB	3/6	4/31	0.109	1/7	6/30	0.771
M5 vs. non-M5 FAB	3/6	4/31	0.109	1/7	6/30	0.771
FAB Type	1/1/1/0/3/0/0/02/1	3/8/4/1/4/2/1/9/0/0	**0.053**	1/3/0/0/1/0/0/1/1/1/8	3/6/5/1/6/2/1/8/1/0	0.420
WHO Type	1/0/0/8	7/11/12/12	**0.049**	1/2/0/5	7/9/2/15	0.770
Primary/Secondary LMA	9/0	25/10	**0.068**	8/0	26/10	**0.090**
Cytogenetic (MRC)	1/7/1	8/18/4	0.579	1/7/0	8/18/5	0.267
Cr 7 Alterations (yes/no)	0/9	4/26	**0.248**	0/8	4/27	0.284
Cr 5 Alterations (yes/no)	1/8	2/28	0.661	1/7	2/29	0.567
Cr 11 Alterations (yes/no)	2/7	5/25	0.703	2/6	5/25	0.560
CD34 Blasts (yes/no)	2/7	20/15	0.062	4/5	18/17	0.709
CD117 Blasts (yes/no)	7/2	22/13	0.400	8/1	21/14	0.103
CD15 Blasts (yes/no)	7/2	12/20	**0.032**	6/3	13/19	0.166
CD11B Blasts (yes/no)	4/5	5/25	**0.083**	2/7	7/23	0.945
CD11C Blasts (yes/no)	2/1	6/2	0.782	2/2	6/1	0.201
CD133 Blasts (yes/no)	2/3	12/16	0.905	2/3	12/16	0.905
CD123 Blasts (yes/no)	2/3	5/18	0.393	2/2	5/19	0.212
HLA DR + BLasts (yes/no)	3/6	19/18	0.408	4/5	18/9	0.236
CD56 Blasts (yes/no)	3/6	19/18	0.048	4/5	18/9	0.236
T-cell markers BLast (yes/no)	1/6	4/13	0.612	2/6	3/13	0.722
Erythroblasts BM	0/4/1	2/8/12	0.312	0/4/3	2/8/10	0.271
Gata-1 Expression (yes/no)	4/5	17/18	0.825	2/6	17/19	0.155
Gata-2 Expression (yes/no)	5/4	17/18	0.709	4/4	18/18	1
EKLF Expression (yes/no)	3/6	18/17	0.332	3/5	18/18	0.522
cMPL Expression (yes/no)	3/6	17/18	0.413	2/6	18/18	0.199
Gata-1 (Median Expression)*	0.127	5.187	0.615	0.107	5.051	0.639
Gata-2 (Median Expression)*	568.23	1273.38	0.759	5000.50	268.84	0.375
EKLF (Median Expression)*	0.0345	23.62	0.593	2.143	22.5	0.660
cMPL (Median Expression)*	3.61	7.01	0.608	4.258	6.768	0.639

Nucleophosmin (NPM1) and FMS-related tyrosine kinase 3 (FLT3) genes. PS, Performance Status. WBC, white blood cell. FAB indicates French American British classification [19] and WHO indicates World Health Organization classification [20]. MRC, cytogenetic classification of Medical Research Cancer [21]. GATA binding protein 1 (GATA1), GATA binding protein 2 (GATA2), Erythroid Krüppel-like Factor (EKLF) and Mieloproliferative Leukemia virus oncogen homology (c-MPL). * All expression ratios are given as 100x target gene/GUS.

**Figure 2:**
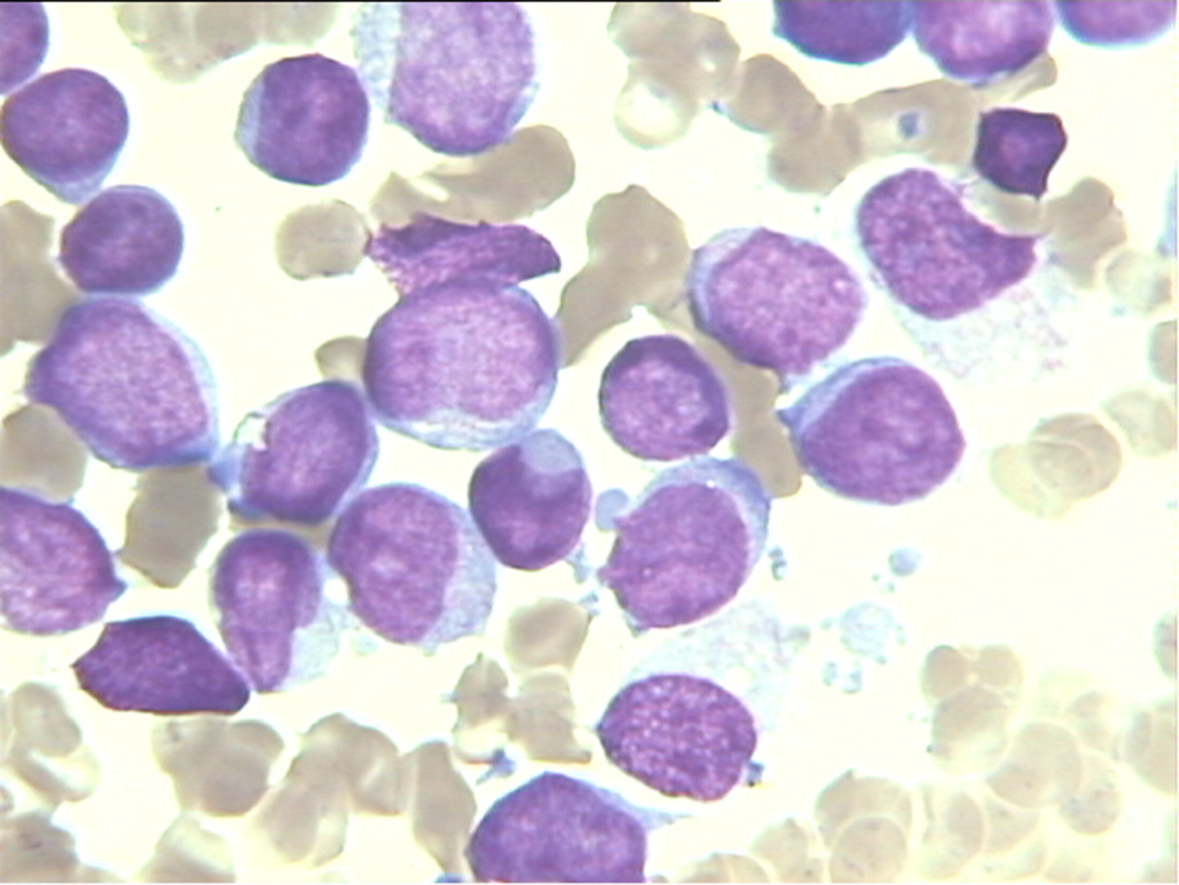
**Morphology of acute myeloid leukemia with cuplike blasts (Wright-Giemsax1000) (McCormick SR, Arch Pathol Lab Med 2010 [**[22]**]).** Only two of nine FLT3-ITD AMLs had cuplike blast morphology, however these patients had high mutation levels.

**FLT3 mutations** were associated with a high percentage of blasts in peripheral blood (66.38 versus 37.78, P = 0.013). FLT3 AML showed a tendency towards association with high WBC (P = 0.051) and *de novo* AML (P = 0.090). FLT3 mutations were more frequent in AML without GATA1 expression (7/20 versus 2/24, P = 0.029). No association with GATA2, EKLF, and cMPL expression (yes/no) was shown. No correlation was observed between GATA1, GATA2, EKLF, and cMPL levels and FLT-3 mutation status (Table 3). Cuplike blast morphology was not associated with FLT3-ITD AMLs, and only 2 of 9 FLT3-ITD positive AMLs had cuplike blast morphology, however both patients had high mutation levels (Figure 2).

### CEBPA mutations and their correlations with GATA1, GATA2, EKLF and cMPL mRNA expression

CEBPA mutations were detected in 4/33 studied cases (12.12%), only one of which was bi-allelic. Four of four CEBPA mutated AMLs were EKLF positive, and 10/29 CEBPA wild-type AMLs and three of CEBPA-mutated, EKLF-positive AMLs were also GATA-2 positive. We should stress that no case with mutated CEBPA was found in the EKLF-negative AML group.

### EKLF mRNA expression was a strong favorable prognostic factor for overall survival and remission duration in AML

**Figure 3:**
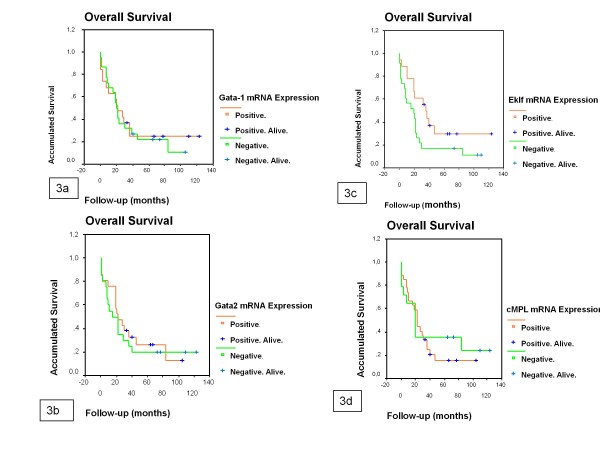
**Univariate analysis found no differences in overall survival (OS) between patients, with and without GATA-1 (3a), GATA-2 (3b), EKLF (3c) and cMPL (3d) mRNA expression.** Patients expressing EKLF had longer OS than those patients not expressing EKLF (median OS was 35.61 months and 19.31 months, respectively, P = 0.0241). GATA binding protein 1 (GATA1), GATA binding protein 2 (GATA2), Erythroid Krüppel-like Factor (EKLF) and Mieloproliferative Leukemia virus oncogen homology (c-MPL).

**Figure 4:**
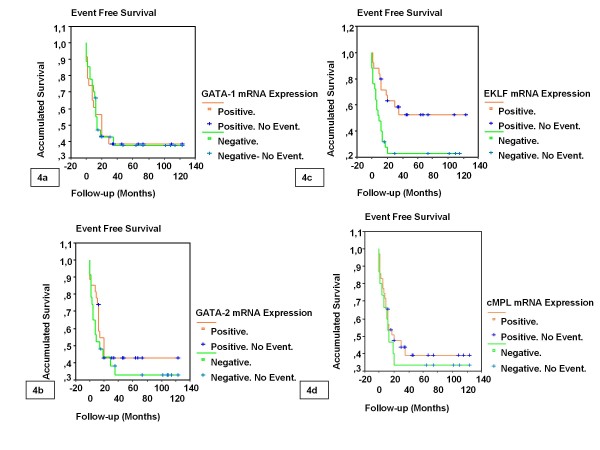
**Univariate analysis found no differences in event free survival (EFS) between patients, with and without GATA-1 (4a), GATA-2 (4b), EKLF (4c) and cMPL (4d) mRNA expression.** Patients expressing EKLF had longer EFS than those patients not expressing EKLF (median EFS was 19.80 months and 8.03 months, respectively, P = 0.0140). GATA binding protein 1 (GATA1), GATA binding protein 2 (GATA2), Erythroid Krüppel-like Factor (EKLF) and Mieloproliferative Leukemia virus oncogen homology (c-MPL).

A prognostic study was performed in 50 patients with AML non-M3, treated with intensive chemotherapy (14 were new patients and 36 previously reported) [17]. The patients studied had a high mortality rate and recurrent leukemia episodes. Univariate analysis found no differences in OS and EFS between patients with and without GATA1, GATA2, and cMPL expression. Patients expressing EKLF had longer OS and EFS than those without EKLF (median OS was 35.61 months and 19.31 months, respectively, P = 0.0241; median EFS was 19.80 months and 8.03 months, respectively, P = 0.0140) (Figures 3 and 4). Patients overexpressing GATA1, GATA2, and cMPL showed no differences in OS and EFS. Patients overexpressing EKLF had a tendency to longer OS and EFS (median OS was 36.69 months and 19.31 months, respectively, P = 0.0682; median EFS was 29.05 months and 8.66 months, respectively, P = 0.0832).

In multivariate analysis considering known prognostic factors (age, gender, type of leukemia, cytogenetic risk group, WBC, FLT3/ITD, and NPM1 mutant status), EKLF expression (yes/no) remained an independent favorable prognostic factor for EFS (HR 0.4504, 95%CI 0.2115-0.9589, P = 0.038) (Table 4). OS was not correlated with any factor when stepwise multivariate analysis was performed. Other studied transcription factors had no influence in OS or EFS in multivariate analysis.

**Table 4:** Multivariate analysis of clinical factors affecting overall survival and event free survival of acute myeloid leukemia (AML) patients

**Variable**	**p**	**Hazard Ratio (CI)**
**a) OVERALL SURVIVAL**		
Age (continuous variable)	0.315	
Gender (male/female)	0.7822	
WBC (continuous variable)	0.9718	
Chromosome 5 alterations	**0.0535**	
Gata-1 expression (negative/positive)	0.4750	
Gata-1 over-expression (zero-low/high)	0.9489	
Gata-2 expression (negative/positive)	0.4750	
Gata-2 over-expression (zer0-low/high)	0.3677	
Eklf expression (negative/positive)	**0.0878**	
Eklf over-expression (zero-low/high)	**0.0772**	
cMPL epression (negative/positive)	0.8023	
cMPL over-expression (zero-low/high)	0.7546	
DIT-FLT3 (WT/mutated)	0.283	
NPM1 mutations (no/yes)	0.234	
Cytogenetic risk (intermediate/good)	0.287	
Cytogenetic risk (adverse/good)	0.139	
**b) EVENT FREE SURVIVAL**		
Age (continuous variable)	0.6064	
Gender (male/female)	0.9844	
WBC (continuous variable)	0.757	
Chromosome 5 alterations	0.6525	
Gata-1 expression (negative/positive)	0.7682	
Gata-1 over-expression (zero-low/high)	0.7405	
Gata-2 expression (negative/positive)	0.9438	
Gata-2 over-expression (zero/low/high)	0.3330	
**Eklf expression (negative/positive)**	**0.0385**	**0.4504 (0.2115-0.9589)**
Eklf over expression (zero-low/high)	0.4693	
cMPL expression (negative/positive)	0.4099	
cMPL over-expression (zero-low/high)	0.8863	
DIT-FLT3 (WT/mutated)	**0.084**	
NPM1 mutations (no/yes)	0.120	
Cytogenetic risk (intermediate/good)	0.704	
Cytogenetic risk (adverse/good)	0.320	

In multivariate analysis considering known prognostic factors (age, gender, type of leukemia, cytogenetic risk group, WBC, FLT3/ITD, and NPM1 mutant status), EKLF expression (yes/no) remained an independent favorable prognostic factor for event free survival (EFS) (HR 0.4504, 95%CI 0.2115-0.9589). Overall survival (OS) was not correlated with any factor when stepwise multivariate analysis was performed. WBC, white blood cell; MRC, cytogenetic classification of Medical Research Cancer [21]. GATA binding protein 1 (GATA1), GATA binding protein 2 (GATA2), Erythroid Krüppel-like Factor (EKLF) and Mieloproliferative Leukemia virus oncogen homology (c-MPL).

## Discussion

We assessed the prognostic significance of mRNA expression of erythroid transcription factors in the context of other cytogenetic and molecular markers in AML. The results demonstrated that GATA1, GATA2, EKLF, and cMPL were variably expressed in newly diagnosed AML patients. EKLF expression was a strong favorable prognostic factor for overall survival and remission duration, as previously reported [17]. We have validated EKLF expression as an independent predictor of outcome in AML.

We attempted to document the dysregulation of erythroid transcription factors and the presence of NPM1 and FLT3 mutations in CN-AML. However, the presence of NPM1 and FLT3 mutations were not associated with cases expressing GATA1, GATA2, EKLF, and cMPL. ITD-FLT3 was more frequent in the AML group that lacked GATA1 expression. ITD mutations inhibit the expression and function of transcription factors involved in myeloid differentiation [11], and GATA1 in hematopoietic cells with ITD mutations can also be downregulated. However, enforced ITD-FLT3 expression in CD34+ cells from cord blood show enhanced erythropoietic potential [23]**.** In our study, FLT3-ITD AML was associated with *de novo* AML, and high WBC and blast cell counts in peripheral blood, as described. Incidentally only two of eight FLT3-ITD AML cases, both with a high FLT3 mutation burden, presented a cuplike blast morphology [22]. NPM1 mutated AML was associated with high WBC and blast cell counts in peripheral blood and bone marrow, a M5 FAB subtype, negative CD34 blasts, and positive CD15 and CD11B blasts, as previously reported [24,25].

EKLF or KLF1 is a member of the zinc family of transcription factors able to bind to GC-rich sequences that are critical regulators of important functions all over the body. It is involved in hematopoiesis, particularly in erythropoiesis, and is also expressed in macrophages [26]. EKLF has surprisingly been associated with tumors such as cancer in the endometrial epithelial cells, and is increased by treatment with tamoxifen or oestrogen [27].

EKLF mRNA expression is correlated with expression levels of other transcription factors, e.g., GATA1, GATA2, and cMPL. Nevertheless the biological characteristics associated with the AML groups expressing GATA1, GATA2, EKLF and cMPL were not completely concordant. GATA1 mRNA expression was more frequent in elderly patients than in AMLs with EKLF expression. GATA2 mRNA expression was more frequent in the group with no abnormalities in Chromosomes 7, 5 and 11. The cMPL mRNA expression group was in accord with the EKLF-expressing AML group, with a low WBC count and FAB M1 or M5 subtypes, but with no influence on prognosis. The causes of these differences are not clear although they could be related to other variables, such as cytogenetic aberrations. Cytogenetic subtype is the most important outcome predictor in AML, but other factors can also play a role. Thus, in a work recently published on genetic aberrations in the leukemogenesis of pediatric AML, genes of the *HOXB* cluster were overexpressed in all patients with a *FLT3*-ITD positive CN-AML, but not in those with a *FLT3*-ITD positive and translocation t (15;17)(q21;q22); a finding in agreement with differences in prognostic relevance between these two subgroups [28]. Le Beau described the loss of GATA1 and EKLF expression in secondary therapy related AML (t-AML) with abnormalities of chromosome 7 but not of chromosome 5 (Group A). On the other hand our patients with similar characteristics showed a loss of GATA1 expression, but conserved EKLF expression and, as Le Beau described, none of these patients had FLT3-ITD mutations. The exact mechanism of dysregulation in these patients remains elusive [29].

In our study GATA2 mRNA expression was not associated with cases with chromosome 7, 5 and 11 abnormalities, and AMLs with minimal differentiation (FAB AML-M0 and CD123 expression). Acute leukaemias are commonly caused by mutations or cytogenetic aberrations that corrupt the transcriptional circuitry of haematopoietic stem/progenitor cells. Recently, Bonadies et al. reported that GATA2 expression is downregulated during the development of AML by abundant GATA motifs in regions of reduced histone acetylation, suggesting an important role in leukemogenic transcriptional reprogramming [30]. Other implications of GATA2 are seen in the neuroblastoma: GATA2, essential for normal SNS development, is downregulated in aggressive neuroblastoma [31].

Association of CEBPA mutations with the upregulation of several genes involved in erythroid differentiation has been reported in a gene and microRNA expression study in CN-AML [32]. CEBPA mutations were associated with upregulation of GATA1, and other genes involved in erythroid differentiation, such as EKLF. In contrast, genes involved in myeloid differentiation, including RUNX1, SPI1, and ID1 were downregulated [32]. CEBPA mutations and NPM1 mutations are mutually exclusive [15,16] and our EKLF-positive AML group was not associated with NPM1 mutated cases. Silencing CEBPA production, an important event in leukemogenesis, could follow the mutation of CEBPA and/or promoter methylation by DNA methyltransferases [13,14]. In our study, EKLF-positive AMLs were secondary leukemias with a low WBC count, CD15, CD11B and T-cell marker expression (such as CD7) in the blast cells, and high erythroblast percentage in the bone marrow. These AML subtypes showed a favorable prognosis.

AMLs with CEBPA mutations show many characteristics also found in the EKLF-positive AML group, such as a high percentage of erythroblasts and favorable outcome. Although the study population was small, we found that all four cases with CEBPA mutations showed EKLF expression, and no case with mutated CEBPA was found in the EKLF-negative AML group. These results support the theory that EKLF expression could be produced through CEBPA dysregulation, either due to mutations or methylation. In this particular group of CEBPA-mutated AMLs, validating EKLF expression as an independent prognostic factor would require a greater number of cases.

## Conclusion

We have further validated EKLF mRNA expression as an independent predictor of outcome in AML not associated with FLT3-ITD and NPM1 mutations. Although additional studies are needed, EKLF expression in AML patients may be related to dysregulated CEBPA. Downregulation of GATA1 expression was found in FLT3-ITD AML or AML with cytogenetic abnormalities of chromosome 7, and GATA-2 downregulation was found in AML with chromosome 5, 7 and 11 abnormalities and undifferentiated AML, but the exact underlying mechanisms remain unknown. It remains to be proved whether transcription factor expression, including EKLF, GATA1 and GATA2, could accurately predict some cytogenetic and molecular subtypes of AML.

## Materials and methods

### Patients

Bone marrow (BM) samples from 65 AML patients referred for treatment at our institution between August 1999 and November 2007 were studied; twenty-four new patients and forty-one previously studied [17]. There were no special criteria for the selection of patients other than available RNA samples at diagnosis, and informed consent for biological studies. Clinical, morphological, cytogenetic, and inmunophenotype characteristics of the patients are summarized in Table 5. According to the UK Medical Research Council (MRC) cytogenetic features criteria [33], 15 patients had favorable cytogenetic features, 31 had an intermediate prognosis, and 12 had bad prognostic features; seven patients were missed for this classification. The study was approved by our Institutional Review Board and all patients gave their consent for blood samples to be further processed.

**Table 5:** Demographic and clinical characteristics of 65 acute myeloid leukemia (AML) patients

**Characteristics**	**Patients**
**Gender** patients (male/female)	65 (37/28)
**Age**, median (range)	51 (16-76)
**WBC** count (X10^9^/L), median (range)	13.4 (0.580-292)
**Hemoglobin** (g/dL), median (range)	9.6 (5.6-12.7)
**Platelet count** (X10^9^/L), median (range)	68 (11-538)
**ECOG PS**, 0/1/2/3/4	17/18/20/9/1
**FAB**	2/5/11/9/6/12/0/2/2/11
10/M1/M2/M3/M4/M5/M6/M7/bilineal/AML-NK/Unclassifiable)	
**WHO**	14/16/3/31
current genetic abnormalities/multilineage dysplasia/therapy-related/not otherwise categorized)	
**De novo** AML/**Secondary** AML	51/14
**Cytogenetic risk group**, (MRC) unfavorable/intermediate/unfavorable/no cytogenetic information)	15/31/12/7
**Blasts** in peripheral blood (%), median (range)	32.5 (2-98)
**Blasts** in bone marrow (%), median (range)	79 (20-100)
**Alterations** in **Chromosome 5/Chromosome 7/Chromosome11/without results**	7 (11.9%)/7 (11.9%)/11 (22.9%)/6
**Blast CD34+** (YES/NO)	33/31
**Blast CD117** (YES/NO)	41/23
**Blast CD56+**YES/NO)	11/54
**Blast CD133+**(YES/NO)	20/27
**Blast CD123+(**YES/NO)	12/23
**Blast HLA-DR+**(YES/NO)	34/21
Patient eligible/not eligible for treatment	50/15

24 were new patients and 41 were previously studied [17]. PS, Performance Status. WBC, white blood cell. FAB indicates French American British classification [19] and WHO indicates World Health Organization classification [20]. MRC, cytogenetic classification of Medical Research Cancer [21].

### Therapy

Fifty patients were treated with chemotherapy; 41 AML non-M3 patients received one or two cycles of Idarubicin/Cytarabine induction treatment and one consolidation cycle with Cytarabine (1 g/m2), and later underwent high dose chemotherapy and autologous stem cell transplantation (15 cases) or allogeneic stem cell transplantation (seven cases). Nine AML M3 patients were treated according to APL99 and APL 2005 PETHEMA protocols [34]. Secondary AML was considered when there was previous chemotherapy treatment or myelodysplastic syndrome. Fifteen non-APL patients over 70 years and with associated co-morbidities were not treated and were excluded from the survival analysis.

### GATA1, GATA2, EKLF and cMPL mRNA expression

GATA1, GATA2, EKLF and cMPL mRNA expression was assessed in all cases as previously described [17]. To assess the quality and quantity of the isolated RNA, as well as the efficiency of cDNA synthesis, each sample was normalized against the expression of beta-glucuronidase (GUS). The construction of standard curves for quantification of the transcription factors and the internal control GUS, and calculation of GATA1, GATA2, EKLF and cMPL copy number normalized to GUS copy number, were performed as previously reported [17]. Quantification was performed by calculating the ratios of the target gene in a standard curve relative to the control gene. All expression ratios are given as 100x target gene/GUS.

### Cytogenetics and mutational analysis of the FLT3, NPM1 and CEBPA genes

Karyotypes were analyzed after G-banding and described according to the International System for Human Cytogenetic nomenclature [35]. Mutational analysis of *FLT3*-ITD was assessed in 34 cases, as previously described [36]. The presence of *NPM1* gene mutations was assessed in 44 cases, as previously described. The presence of *CEBPA* gene mutations was assessed in 33 cases, as previously described [37].

### Definition of clinical end points

Complete remission (CR) was defined as recovery of morphologically normal BM and blood counts (i.e., neutrophils 1,500/μL and platelets 100,000/μL), and no circulating leukemic blasts or evidence of extramedullary involvement. EFS was defined as the interval from the date of study until removal from the study because of failure to achieve CR, relapse, or death as a result of any cause (whichever occurred first). Patients alive without relapse were censured, whereas those who died without relapse were counted as a competing cause of failure.

### Statistical methods

The following variables collected at diagnosis were included in the database: gender, age (both as a continuous variable and grouping patients over and under 55 years of age), Eastern Cooperative Oncology Group performance status (ECOG PS), white blood cell (WBC) count, platelet count, hemoglobin (Hb) level, percentage of blast cells in BM and in PB (over and under 70% blast cells), percentage of erythroblast in BM (in three groups 0–5%/5– 15%/>15%), immunophenotypes, cytogenetics, and World Health Organization (WHO) [38] and French American British (FAB) [39] classifications.

Karyotype findings were grouped according to the Medical Research Council (MRC) classification [33] into: (1) favorable cytogenetic markers; (2) intermediate (no abnormalities, +8, 11q23, del [9q], +22, and other abnormalities not included in the other groups); and (3) adverse cyto-genetics (complex, - 7, abnormalities in 3q, del [5q] and −5). Seven patient karyotypes were unavailable.

Analysis of GATA1, GATA2, EKLF, and c-MPL mRNA expression and overexpression were done with FAB subtypes AML and grouping: M2 against non-M2; M3 against non-M3 and M5 against non-M5 (these being the great majority of cases studied). Expression was defined as detection of a transcription factor versus its absence. Overexpression was defined as expression values over the median: Gata1/GUS > 0.057% for GATA1; Gata2/GUS > 0.272% for GATA2; EKLF/GUS > 0.177% for EKLF, and c-Mpl/GUS > 0.839% for c-MPL.

Survival studies were carried out in the patients treated with chemotherapy. Overall survival (OS) was calculated from the first day of therapy to death, and Event Free Survival (EFS) to no response, relapse, or death. Kaplan-Meier life tables were constructed for survival data and were compared by means of the log-rank test, with a census of the surviving patients taken in July, 2007. Median follow-up was 19.4 months (range 0.5–62.1 months). Results with a P value of less than 0.05 were considered significant.

A conditional logistic-regression model was used to analyze associations between baseline characteristics and GATA1, GATA2, EKLF, and cMPL mRNA expression, or the NPM1, FLT3 and CEBPA mutations. A Cox model was used to identify prognostic variables.

## Abbreviations

AML, Acute Myeloid Leukemia; BM, Bone Marrow; cMPL gene, Myeloproliferative Leukemia virus oncogen homology gene; FLT3 gene, FMS-like Tyrosine Kinase 3 gene; NPM1 gene, Nucleophosmin 1 gene; CEBPA gene, CCAAT/enhance-binding protein α gene; EKLF, Erythroid Krüppel-like Factor; GATA1, GATA binding protein 1; GATA2, GATA binding protein 2; RUNX1, Runt Box1; EFS, Event Free Survival; OS, Overall Survival; ECOG PS, Eastern Cooperative Oncology Group Performance status; FAB, French-American-British Classification; WHO, World Health Organization Classification; CN-AML, Cytogenetically normal acute myeloid leukemia; CD, Cluster of differentiation; MRC, Medical Research Center.

## Competing interests

The authors declare that they have no competing interests.

## Authors’ contributions

RA, JML and FG designed the project. RA and JML performed molecular analysis. All authors drafted the manuscript. All authors read and approved the final manuscript.

## Domains

Oncology, Hematology, Development & Embryology, Genes & Genomics, Biochemistry & Molecular Biology

## Synopsis

Disturbance of mRNA expression of erythroid transcription factors, such as EKLF, GATA1 or GATA2, has been shown in patients with acute myeloid leukemia (AML), but no direct link to the mutations responsible for leukemogenesis has been demonstrated. We studied the role of these transcription factors in AML in the context of other prognostic molecular markers, such as FLT3-ITD, NPM1 and CEBPA mutations.
